# Purification and Properties of Polygalacturonase Produced by Thermophilic Fungus *Thermoascus aurantiacus* CBMAI-756 on Solid-State Fermentation

**DOI:** 10.1155/2013/438645

**Published:** 2013-09-12

**Authors:** Eduardo da Silva Martins, Rodrigo Simões Ribeiro Leite, Roberto da Silva, Eleni Gomes

**Affiliations:** ^1^Laboratório de Microbiologia, Universidade do Estado de Minas Gerais (UEMG), Avenida Prof. Mario Palmerio 1000, 38200-000 Frutal, MG, Brazil; ^2^Faculdade de Ciências Biológicas e Ambientais (FCBA), Universidade Federal da Grande Dourados (UFGD), Rodovia Dourados-Itahum, Km 12, 79804-970 Dourados, MS, Brazil; ^3^Laboratório de Bioquímica e Microbiologia Aplicada, Instituto de Biociências, Universidade Estadual Paulista (UNESP), Rua Cristovão Colombo 2265, Jd. Nazareth, 15054-000 São José do Rio Preto, SP, Brazil

## Abstract

Polygalacturonases are enzymes involved in the degradation of pectic substances, being extensively used in food industries, textile processing, degumming of plant rough fibres, and treatment of pectic wastewaters. Polygalacturonase (PG) production by thermophilic fungus *Thermoascus aurantiacus* on solid-state fermentation was carried out in culture media containing sugar cane bagasse and orange bagasse in proportions of 30% and 70% (w/w) at 45°C for 4 days. PG obtained was purified by gel filtration and ion-exchange chromatography. The highest activity was found between pH 4.5 and 5.5, and the enzyme preserved more than 80% of its activity at pH values between 5.0 and 6.5. At pH values between 3.0 and 4.5, PG retained about 73% of the original activity, whereas at pH 10.0 it remained around 44%. The optimum temperature was 60–65°C. The enzyme was completely stable when incubated for 1 hour at 50°C. At 55°C and 60°C, the activity decreased 55% and 90%, respectively. The apparent molecular weight was 29.3 kDa, *K*_*m*_ of 1.58 mg/mL and *V*_max_ of 1553.1 **μ**mol/min/mg. The presence of Zn^+2^, Mn^+2^, and Hg^+2^ inhibited 59%, 77%, and 100% of enzyme activity, respectively. The hydrolysis product suggests that polygalacturonase was shown to be an endo/exoenzyme.

## 1. Introduction

Pectinases are a heterogeneous group of enzymes that hydrolyze the pectic substances present in plant material. The classification of pectinases is based on their mode of attack on the galacturan backbone of the pectin molecule, on specificity by substrate, or according to region of molecule where it acts [1].

The polygalacturonases catalyze the hydrolysis of glycosidic *α*-1-4 linkages in pectic acid and are of two types: endo-polygalacturonases (endo PG, EC 3.2.1.15), which act by hydrolysis of internal glycosidic bonds *α*-1-4 of polygalacturonic acid at random form, resulting in molecule depolymerization with release of oligogalacturonic acids, and exo-polygalacturonases (exo PG, EC 3.2.1.67) which hydrolyse alternate *α*-1-4 glycosidic linkages of polygalacturonic acid from the nonreducing end, releasing unsaturated mono- or digalacturonic acids [2, 3].

This group of enzymes has been widely used in the food industry process such as clarification and viscosity reduction of fruit juices, preliminary treatment of grape juice for wine industries, tomato pulp extraction, oil extraction, and tea fermentation and in the textile industry in fibers degumming [4, 5].

In the literature, it has been reported that the type of fermentation influences the enzymes properties, such as thermostability and tolerance to pH variations [6, 7]. According to Acuña-Argüelles et al. [8], pectinases obtained by *Aspergillus oryzae* cultivation in solid-state fermentation (SSF) were more resistant to pH and temperature changes compared to those obtained by submerged fermentation (SmF). Moreover, Martin et al. [9] reported that polygalacturonase obtained by *Thermomucor indicae-seudaticae* showed higher thermostability in SmF than that in SSF. Thus, the present study aimed to purify polygalacturonase produced by thermophilic fungus *Thermoascus aurantiacus* in solid-state fermentation and compare its properties with those of the purified polygalacturonase produced by the same fungus in SmF, in a work reported by Martins et al. [10].

## 2. Materials and Methods

### 2.1. Microorganism

Thermophilic fungus *Thermoascus aurantiacus *CBMAI756 was used. The strain is deposited in the Coleção Brasileira de Microrganismos de Indústria e Meio Ambiente-CBMAI, UNICAMP, Campinas, SP.

### 2.2. Media, Cultivation of Microorganism, and PG Production

SSF was carried out using a 250 mL Erlenmeyer flask containing 5 g of sterilized mixture of sugar cane bagasse and orange bagasse in proportions of 30 and 70% (w/w). The material was inoculated with 5 mL of micelial suspension (14.5 mg dry micelial mass/g dry substrate) which was obtained from a 4-day agar slant culture suspended in sterile distilled water. 

After inoculation, 10 mL of nutrient solution composed of 0.1% NH_4_NO_3_, 0.1% NH_4_H_2_PO_4_, 0.1% MgSO_4_·7H_2_O at pH 5.0 was added to each of the several flasks. The final moisture content of the medium was approximately 70%. Cultivation was carried out at 45°C for 4 days. At 24 h intervals, the material corresponding to one Erlenmeyer flask was mixed with 40 mL distilled water, stirred for 40 min, filtered under vacuum, and centrifuged. The supernatant was used as crude enzyme solution.

### 2.3. Enzyme Activity Measurements

Exo-polygalacturonase (exo-PG) activity was assayed in a mixture containing 0.4 mL of 1% of citrus pectin solution (26% esterified—Sigma) in 0.2 M sodium acetate buffer (pH 5.5) and 0.1% of crude enzyme solution at 60°C for 10 min. The number of reducing groups, expressed as galacturonic acid released by enzymatic action, was quantified by the DNS method [11]. One unit of enzyme activity (U) was defined as the amount of enzyme releasing 1 *μ*mol of galacturonic acid per minute under the assay conditions.

Endo-PG activity was measured viscosimetrically by adding 2 mL of crude enzyme to 6 mL of 0.2 M acetate-NaOH buffer (pH 5.5) containing 3% of low-esterified citrus pectin (Sigma). The reaction mixture was incubated at 60°C for 15 min, and its viscosity was determined with a basic viscosimeter (Fungilab). One unit of enzyme activity was defined as the amount of enzyme that reduced the initial viscosity by 50% per minute.

### 2.4. Enzyme Purification

150 mL of crude enzyme extract was dialyzed against 10 mM acetate buffer, pH 4.0, overnight. After dialysis, it was lyophilized and resuspended in 20 mL of 10 mM acetate buffer, pH 4.0.

Gel filtration chromatography with Sephadex G-75 column (90.0 cm × 2.5 cm—Pharmacia) was used, and the elution occurred with 20 mM acetate buffer pH 4.0 at a flow rate of 16.8 mL/h. The PG activity (DNS assay method) and protein content of each tube were determined. The fractions containing the peak of enzyme activity were joined to the next step purification process in ion-exchange column.

For ion-exchange chromatography, SP Sepharose column (20.0 cm × 2.5 cm—Aldrich) was used and the elution was made with same acetate buffer and flow at salt gradient from 0 to 1.1 M NaCl. The solution containing PG peak activity was desalted by dialysis against 10 mM acetate buffer, pH 4.0, at 4°C, overnight.

### 2.5. Analytical Electrophoresis

The molecular weight of the purified enzyme was determined by SDS-PAGE in a Mini Protean II apparatus (10 × 8 cm) (Biorad). Electrophoresis was carried out in polyacrylamide gel, consisting of a 4% (w/v) stacking gel and 10% (w/v) resolving gel in Tris/glycine buffer (pH 8.3), by the method of Laemmli [12]. The molecular weight marker (Sigma M6539, 6.5–180 kDa) was used. The protein band was visualized by silver staining.

Analytical isoelectric focusing was performed in an Ettan IPGphor II Isoelectric Focusing system (Amersham) by electrophoresis in a 7.5% polyacrylamide gel (14 × 15 cm) containing 5% Pharmalyte (pH 3.0–10.0) (purchased from Amersham Bioscience). The gel was silver-stained to reveal protein.

### 2.6. Protein Determination

Protein concentration was determined in the concentration of 10–100 *μ*g/mL by the microassay method of Bradford [13], using bovine serum albumin (BSA) as the standard.

### 2.7. Enzyme Characterization

For characterizing the PG activity, the DNS assay method was used. Optimal activity of purified PG was assayed as a function of pH, in 200 mM acetate buffer (pH 3.0–5.5), citrate-phosphate (pH 6.0–7.0), Tris-HCl (pH 7.5–8.5), and glycine-NaOH (pH 9.0–11.0), at 60°C with 2% low-esterified pectin (2%) as substrate. The pH stability of PG was evaluated by incubation of enzymatic solution in 0.1 M buffer solutions acetate (pH 3.0 to 5.5), citrate-phosphate (pH 6.0–7.0), Tris-HCl (pH 7.5–8, 5), and glycine-NaOH (pH 9.0–11.0), in the absence of pectin, for 24 hours. After this period, an aliquot was taken to measure residual activity under conditions of optimum pH and temperature. 

The effect of temperature on enzymatic activity was evaluated by incubation of reaction mixture at temperatures from 40°C to 80°C for 10 minutes, at optimum pH. The thermal stability was determined by measuring the residual activity of the enzyme after 1 h of incubation, in absence of substrate, at temperatures between 10 and 90°C. After this period, samples were taken to assay enzyme activity under conditions of optimum pH and temperature.

To determine substrate specificity, solution of polygalacturonic acid, citrus pectin with 26% and 92% degree of esterification, and apple pectin (Sigma) were used as substrates at 2.0% in 0.2 M acetate buffer. The reaction was conducted in optimal conditions of enzyme activity.

The influence of metallic ions on PG activity was evaluated by incubation of enzyme in the presence of different ionic solutions at 2 mM (Fe^+3^, Ag^+^, Ca^+2^, Mg^+2^, Mn^+2^, Zn^+2^, K^+^, and Hg^+2^) and EDTA at final concentration in the reaction medium of 2 mM. After 10 min. incubation at 4°C, the residual activity was measured under conditions of optimum enzyme activity.

The Michaelis constant (*K*_*m*_) and *V*_max⁡_ values were determined from Lineweaver-Burk plots of enzyme activity measured with citrus pectin with 26% degree of esterification (Sigma) as substrates, at concentrations between 0.25 and 1.25% at optimum pH and temperature. The results were plotted with the program Grafit 5.0. 

The hydrolysis products of 26% esterified citrus pectin and trigalacturonic acid were analyzed by chromatography on Whatman no. 1 paper, using as solvent a mixture of n-butanol, acetic acid, and water at a ratio of 5 : 3 : 2, respectively, and as developing solvent acetone and silver nitrate (to saturation), washed with alcoholic hydroxide silver for visualization of the bands. The mono-, di-, and trigalacturonic acids (Sigma) were used as standards. 

## 3. Results and Discussion

### 3.1. Purification of PG

**Figure 1:**
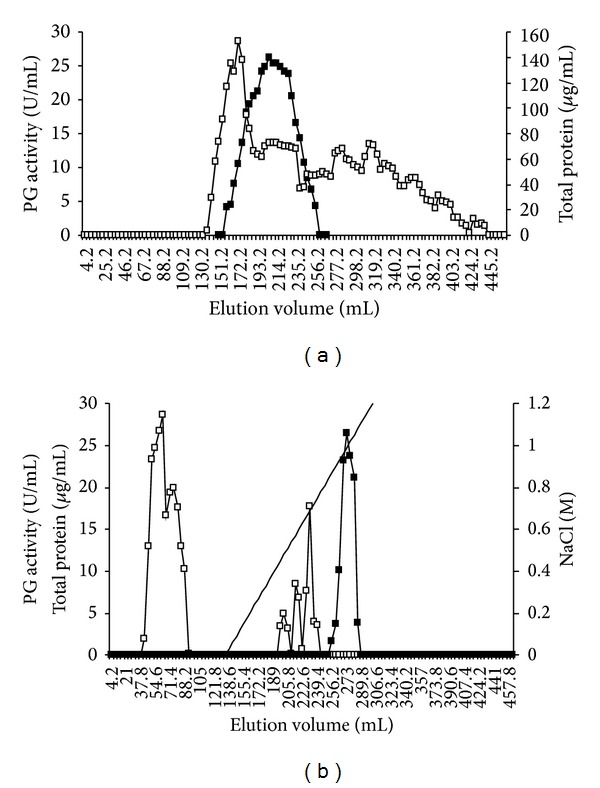
Elution of PG activity from chromatography columns previously equilibrated with 20 mM acetate buffer, pH 4.0: (a) Sephadex G-75 column (3.0 × 80 cm) and (b) SP Sepharose (2.5 × 20 cm) eluted with a NaCl gradient (0–1.1 M). -■- PG activity; -□- protein; — NaCl gradient.

The crude enzyme solution obtained by fungus culture on solid-state fermentation applied on Sephadex G-75 gel column showed only one peak of enzyme activity, which was detected between 160.0 mL and 256.2 mL (Figure 1(a)). This step resulted in an increasing in the specific activity from 60.0 U/mg to 331.6 U/mg protein, in 5.2-fold enzyme purification and 58.8% yield (Table 1).

In the second step, 50 mL of enzymatic extract was applied on ion-exchange chromatography, using 20 mM acetate-NaOH buffer, at pH 4.0. Two protein peaks were observed from the elution volumes of 42.0 mL and 88.2 mL before the start of the salt gradient and three between 0.15 M and 0.7 M NaCl. Polygalacturonase was eluted at 0.9 M salt concentration (Figure 1(b)). The specific activity increased from 331.6 U/mg to 5351.5 U/mg protein, with 89.2-fold enzyme purification and 14.2% yield (Table 1).

**Figure 2:**
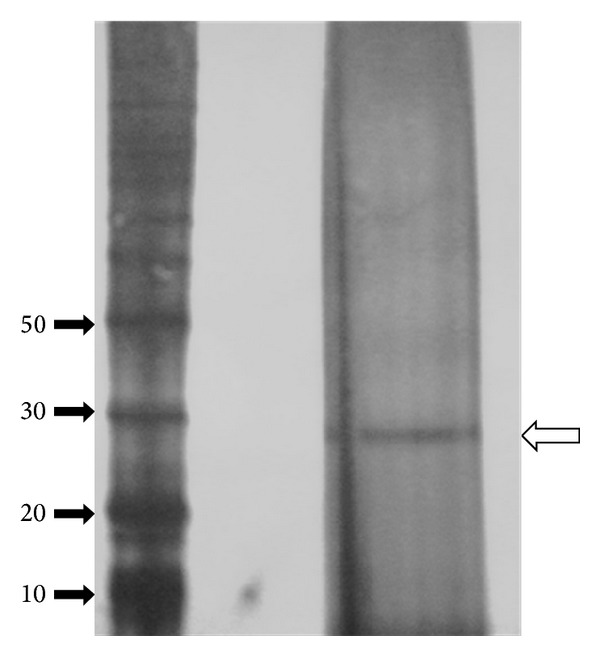
SDS-PAGE of purified PG from gel filtration and ion-exchange chromatography. The numbers of the left indicate the positions of molecular weight markers in kDa.

The samples application on gel electrophoresis indicated that the enzyme was purified to homogeneity and had molecular weight of 29.3 kDa (Figure 2), similar to PG produced in submerged fermentation presented by Martins et al. [10]. PGs with very similar molecular weight were also described by Saito et al. [14] (29.7 kDa) studying the fungus *Rhizopus oryzae* and Niture and Pant [15] (30.6 kDa) studying the fungus *Fusarium moniliforme *in solid-state fermentation.

### 3.2. Enzyme Properties

**Figure 3:**
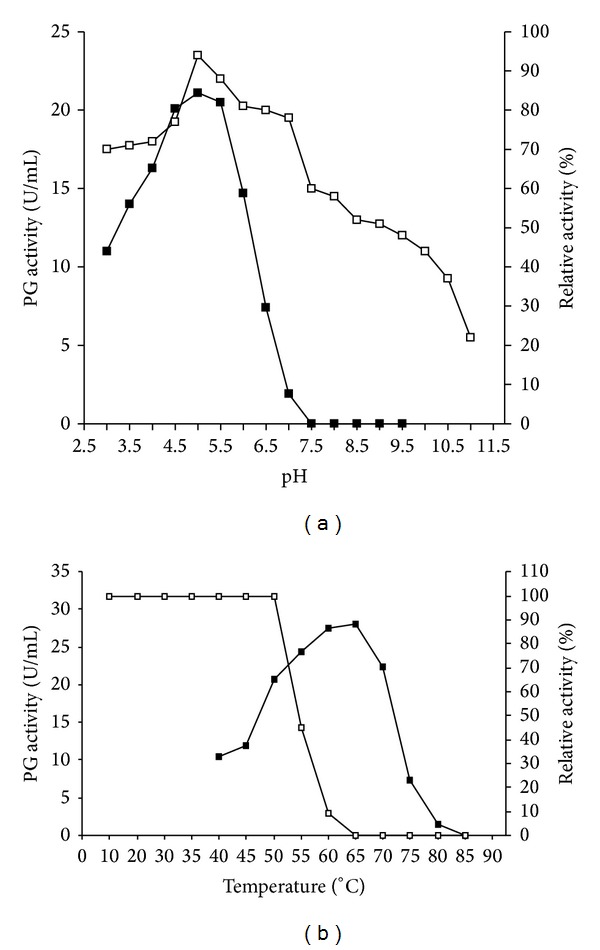
Effect of pH (a) and temperature (b) on the PG activity and stability. -■- activity in the presence of substrate expressed in U/mL; -□- stability in absence of substrate expressed in % of the original activity.

The highest activity was found between pH 4.5 and 5.5 (Figure 3(a)) and when maintained for 24 h in different pH values, in the absence of substrate, the enzyme preserved more than 80% of its activity at pH values between 5.0 and 6.5. In more acidic pH values (3.0 to 4.5), the enzyme retained about 73% of the original activity, whereas at pH 10.0 it remained around 44% (Figure 3(a)).

The response to the effects of pH presented by the enzyme produced in SSF was quite different from that observed for the same enzyme produced in SmF presented by Martins et al. [10], which showed maximum activity between pH 5.5 and 6.0 and was stable in a very narrow pH range (between 5.0 and 7.5). However, it is similar to the data reported by Siddiqui et al. [16], with the PG produced by *Rhizomucor pusillus *in solid-state fermentation, which showed optimum pH of 5.0 and a wide range of pH stability.

Regarding the influence of temperature on the enzyme activity, it was observed that the PG was most active between 60–65°C, with a reduction of about 75% activity at 75°C. When incubated for 1 hour at different temperatures, in the absence of substrate, a pure PG maintained 100% of the original activity at 50°C. At 55°C and 60°C, the activity decreased 55% and 90%, respectively, whereas at 70°C the enzyme was denatured (Figure 3(b)). 

This result is similar to the thermostability of PG produced in solid-state fermentation by *Rhizomucor pusillus*, which showed 100% stability at 50°C for 1 hour, but at 60°C its stability decreased [16]. Comparison of these data with those for enzyme produced in submerged fermentation related by Martins et al. [10] indicates that the enzyme obtained from SSF was less thermostable, since the enzyme SmF retained 25% and 10% at 60°C and 70°C, respectively.

**Figure 4:**
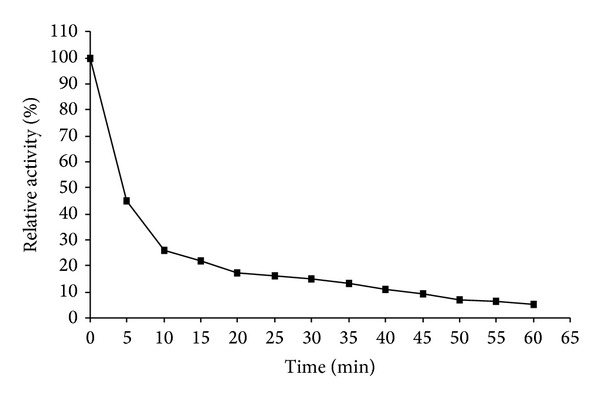
Stability of PG at 60°C in the absence of substrate expressed in % of the original activity.

The half-life of PG at 60°C was approximately 5 minutes (Figure 4), even lower than that found for the PG obtained in SmF [10], in which half -life was approximately 10 minutes.

There are few reports in the literature on the influence of temperature on the pectinases activity from thermophilic fungi. Kaur et al. [17] reported the partial purification and characterization of a polygalacturonase produced by thermophilic fungus *Sporotrichum thermophile* in submerged fermentation, which showed optimum temperature of 55°C. The purified enzyme by the fungus *Acrophialophora nainiana* showed greater activity at 60°C [18]. 

Kumar and Palanivelu [19] reported that purified PG of the thermophilic fungus *Thermomyces lanuginosus* retained only 4% of activity at 60°C and was completely inhibited when exposed for 1 hour at 70°C.

Pectinases obtained by *Aspergillus oryzae* cultivation in solid-state fermentation (SSF) were more resistant to pH and temperature changes compared to those obtained by submerged fermentation (SmF) [8]. Moreover, Martin et al. [9] reported that polygalacturonase obtained by *Thermomucor indicae-seudaticae* showed higher thermostability in SmF than that in SSF.

In relation to substrate preference, PG showed the highest activity with 26% esterified citrus pectin (Table 2) similar to that observed for the enzyme obtained from SmF by Martins et al. [10], indicating that this fungal strain has a polygalacturonase with a preference for hydrolyzing low-esterified pectin.

To evaluate the influence of ions on PG activity, it was observed that ion Ag^+^ caused 18% decrease in PG activity (Table 3), while the same enzyme produced in SmF by Martins et al. [10] had a 10% increase in its activity in their presence.

The ions Mg^+2^, Zn^+2^ Mn^+2^, and EDTA also partially inhibited the enzyme activity, with decrease of 24%, 59%, 77%, and 27%, respectively (Table 3), similar to the results obtained for PG of SmF [10].

The ion Mg^+2^ (2 mM) also inhibited about 50% enzyme activity of polygalacturonase produced by *Fusarium oxysporum* in SmF [20]. On the other hand, the PG activity produced by *Sporotrichum thermophile* was inhibited by 78% with this ion at 1 mM [17]. Regarding Zn^+2^, similar results were found for other fungal PGs, which also suffered inhibition when exposed to this ion. The polygalacturonase produced by *Thermomyces lanuginosus* was inhibited by 53% [19], while that produced by *Sporotrichum thermophile* was inhibited by around 50% with this ion at 1 mM [17].

The PG activity was completely inhibited in the presence of Hg^+2^ (Table 3), similar to that observed for PG obtained by SmF [10]. Inhibition by thiol group blocking agents such as Hg^+2^ suggests a possible involvement of this group in the enzyme active site. Three polygalacturonases purified from *Aspergillus carbonarius* were also inhibited by this ion, even at very low concentration (0.02 mM) [21]. The effect of ions in oxidative enzyme stability can be attributed to cysteine oxidation, which causes the formation of intramolecular and intermolecular disulfide bridges or rearranging these links, leading to the formation of sulfuric acid, resulting in enzyme structural change [22].

The *K*_*m*_ of the PG was 1.58 mg/mL and *V*_max⁡_ was 1553.1 *μ*mol/min/mg protein. The enzyme obtained from SmF showed *K*_*m*_ of 0.62 mg/mL, and *V*_max⁡_ of 2433.2 *μ*mol/min/mg [10]. These values indicate that the enzyme from solid-state fermentation (SSF) showed lower affinity for the substrate compared with that of SmF, since the value of *K*_*m*_ was high.

According to Mohamed et al. [23], the *K*_*m*_ of fungal polygalacturonases generally ranges from 0.12 to 6.7 mg/mL. These authors purified two polygalacturonases from *Trichoderma reesei* which had *K*_*m*_ values of 0.15 mg/mL (PG I) and 0.93 mg/mL (PG II). Kaur et al. [17] purified the PG of the fungus *Sporotrichum thermophile*, with a *K*_*m*_ of 0.416 mg/mL. The PG purified from the thermophilic fungus *Acrophialophora nainiana* showed a *K*_*m*_ quite high (4.22 mg/mL), indicating low affinity for citrus pectin [18].

The isoelectric point of the PG obtained from SSF was 6.6, a value different from that observed for enzyme produced in SmF which was 7.8 [10]. These values indicate the presence of higher amount of negatively charged residues in the amino acids of PG obtained by SSF, which showed lower PI.

The pI values of the two PGs obtained by *T. aurantiacus* are similar to those of other fungal strains described in the literature. García Maceira et al. [20] purified a PG of *Fusarium oxysporum*, which showed pI 7.0. Cabanne and Donèche [24] reported the purification of two pectinases, an endo-PG with pI 7.8 and an exo-PG with pI 8.0. Niture and Pant [15] described the purification of a polygalacturonase with pI 8.6. The pI is a characteristic that varies widely between pectinases obtained by different microorganisms and even between different strains of the same fungal species. This fact can be illustrated by the results found by Pashkoulov et al. [25], who reported the purification and characterization of PGs isolated from five different strains of *Botrytis cinerea*. The authors observed that the enzyme from each strain showed different isoelectric points, ranging between 5.0 and 9.0.

**Figure 5:**
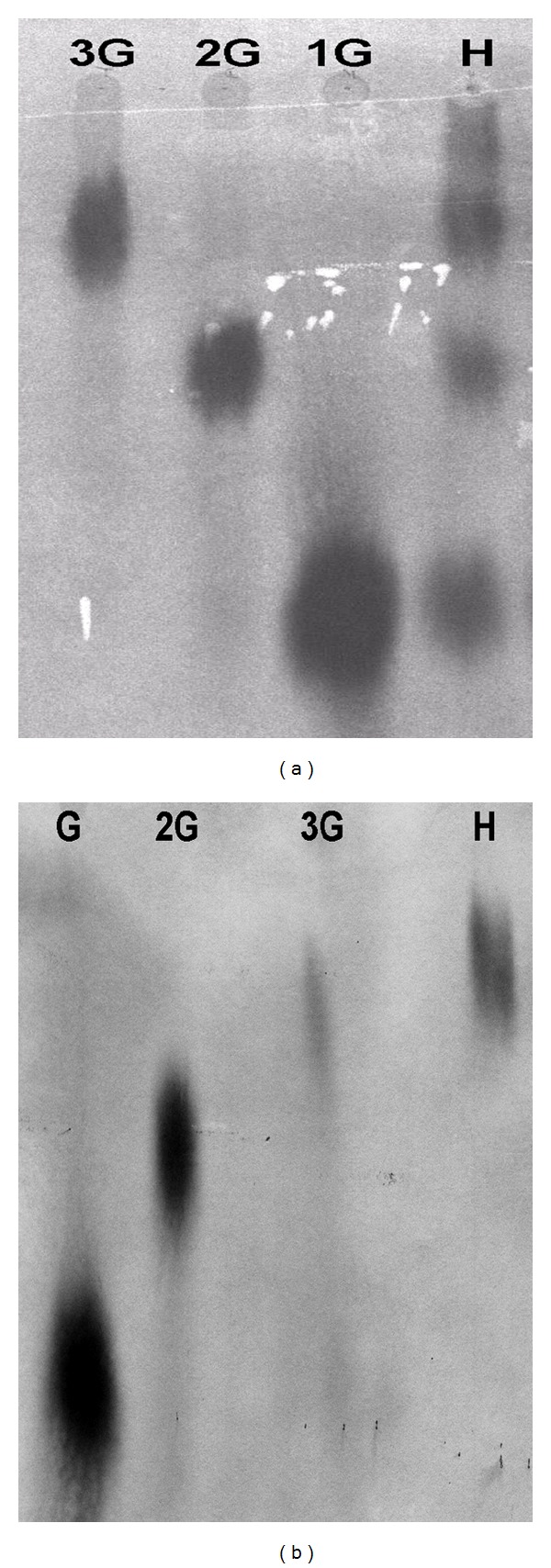
Paper chromatographic analysis of hydrolysis products of PG from *Thermoascus aurantiacus*. (a) Acting on 1% (w/v) citrus pectin at 65°C, for 10 min. (b) Acting on 1% (w/v) trigalacturonic acid at 65°C, for 10 min. 3G: trigalacturonic acid; 2G: digalacturonic acid; 1G: galacturonic acid; H: enzyme hydrolyzed pectin.

After incubation for 5 min at 65°C in 1% citrus pectin 26% DE methoxylation, the polygalacturonase released a mixture of mono-, di-, tri-, and oligogalacturonic acids (Figure 5(a)) and was not able to hydrolyze the trigalacturonic acid (Figure 5(b)). 

The PG activity measured by viscosimetric assay method (specific activity of 3542.8 U/mg) reduced afforded 56% of viscosity of 1% citrus pectin in 10 min. These results indicated an endo-PG activity. On the other hand, di and mono-galacturonates were released at the initial stages of the incubation period, suggesting that PG degraded the substrate by multiple attacks.

This profile was very similar to that observed for the SmF enzyme by Martins et al. [10], which was considered an enzyme with endo/exoactivity. Similar results were also found by Contreras Esquivel and Voget [26], who observed this same attack mode with the polygalacturonase produced by *Aspergillus kawachii *in SmF. This PG was also unable to degrade digalacturonic and trigalacturonic acids but released unsaturated mono-, di-, tri-, and oligogalacturonic acids, indicating that it presents action in multiple attacks.

## 4. Conclusions

The polygalacturonase obtained by thermophilic fungus *Thermoascus aurantiacus* CBMAI756 in solid-state fermentation showed higher tolerance to variations of pH compared with PG produced in SmF by the same fungus, though that was less thermostable than SmF enzyme. Other enzymes features, such as isoelectric point, *K*_*m*_, and influence of some ions in the activity, were also different in relation to the fermentation process employed.

## Figures and Tables

**Table 1:** Purification of the PG produced by *Thermoascus aurantiacus* in SSF.

Step	Volume (mL)	Total activity (U)	Total protein (mg)	Specific activity (U/mg protein)	Purification fold	Yield (%)
Lyophilized extract	8.00	1800.00	30.00	60.00	1.00	100.00
Sephadex G-75	54.00	1058.40	3.19	331.60	5.53	58.80
SP Sepharose	12.60	255.80	0.0478	5351.50	89.19	14.20

**Table 2:** Substrate specificity of PG from *T. aurantiacus *produced on SSF.

Substrate	PG activity (U/mL)
Citrus pectin 26% DE methoxylation	22.5
Citrus pectin 92% DE	9.6
Apple pectin	3.6
Polygalacturonic acid	2.4

**Table 3:** Influence of ions on the PG activity.

Ion	Residual activity (%)
Control	100
Fe^+3^	94
Ag^+^	82
Ca^+2^	94
Mg^+2^	76
Mn^+2^	23
Zn^+2^	41
K^+^	100
Hg^+2^	0
EDTA	73

## References

[1] Kashyap DR, Vohra PK, Chopra S, Tewari R (2001). Applications of pectinases in the commercial sector: a review. *Bioresource Technology*.

[2] Rombouts FM, Pilnik W, Rose AH (1980). Pectic enzymes. *Economic Microbiology*.

[3] Nakkeeran E, Subramanian R, Umesh-Kumar S (2010). Purification of polygalacturonase from solid-state cultures of *Aspergillus carbonarius*. *Journal of Bioscience and Bioengineering*.

[4] Jayani RS, Saxena S, Gupta R (2005). Microbial pectinolytic enzymes: a review. *Process Biochemistry*.

[5] Uenojo M, Pastore GM (2007). Pectinases: aplicações industriais e perspectivas. *Química Nova*.

[6] Rodríguez-Couto S, Sanromán A (2005). Application of solid-state fermentation to ligninolytic enzyme production. *Journal of Food Engineering*.

[7] Martins EDS, Leite RSR, da Silva R, Gomes E (2012). Production and characterization of polygalacturonase from thermophilic *Thermoascus aurantiacus* on submerged fermentation. *Annals of Microbiology*.

[8] Acuña-Argüelles ME, Gutierrez-Rojas M, Viniegra-Gonzalez G, Favela-Torres E (1995). Production and properties of three pectinolytic activities produced by *Aspergillus niger* in submerged and solid-state fermentation. *Applied Microbiology and Biotechnology*.

[9] Martin N, Guez MA, Sette LD, Da Silva R, Gomes E (2010). Pectinase production by a Brazilian thermophilic fungus *Thermomucor indicae-seudaticae* N31 in solid-state and submerged fermentation. *Mikrobiologiia*.

[10] Martins ES, Silva D, Leite RSR, Gomes E (2007). Purification and characterization of polygalacturonase produced by thermophilic *Thermoascus aurantiacus* CBMAI-756 in submerged fermentation. *International Journal of General and Molecular Microbiology*.

[11] Miller GL (1959). Use of dinitrosalicylic acid reagent for determination of reducing sugar. *Analytical Chemistry*.

[12] Laemmli UK (1970). Cleavage of structural proteins during the assembly of the head of bacteriophage T4. *Nature*.

[13] Bradford MM (1976). A rapid and sensitive method for the quantitation of microgram quantities of protein utilizing the principle of protein dye binding. *Analytical Biochemistry*.

[14] Saito K, Takakuwa N, Oda Y (2004). Purification of the extracellular pectinolytic enzyme from the fungus *Rhizopus oryzae* NBRC 4707. *Microbiological Research*.

[15] Niture SK, Pant A (2004). Purification and biochemical characterization of polygalacturonase II produced in semi-solid medium by a strain of *Fusarium moniliforme*. *Microbiological Research*.

[16] Siddiqui MA, Pande V, Arif M (2012). Production, purification and characterization of polygalacturonase from *Rhizomucor pusillus* isolated from decomposting orange peels. *Enzyme Research*.

[17] Kaur G, Kumar S, Satyanarayana T (2004). Production, characterization and application of a thermostable polygalacturonase of a thermophilic mould *Sporotrichum thermophile* Apinis. *Bioresource Technology*.

[18] Celestino SMC, Maria de Freitas S, Javier Medrano F, Valle de Sousa M, Filho EXF (2006). Purification and characterization of a novel pectinase from *Acrophialophora nainiana* with emphasis on its physicochemical properties. *Journal of Biotechnology*.

[19] Kumar SS, Palanivelu P (1999). Purification and characterization of an extracellular polygalacturonase from the thermophilic fungus, *Thermomyces lanuginosus*. *World Journal of Microbiology and Biotechnology*.

[20] García Maceira FI, Di Pietro A, Roncero MIG (1997). Purification and characterization of a novel exopolygalacturonase from *Fusarium oxysporum* f.sp. lycopersici. *FEMS Microbiology Letters*.

[21] Anjana Devi N, Appu Rao AG (1996). Fractionation, purification, and preliminary characterization of polygalacturonases produced by *Aspergillus carbonarius*. *Enzyme and Microbial Technology*.

[22] Vieille C, Zeikus GJ (2001). Hyperthermophilic enzymes: sources, uses, and molecular mechanisms for thermostability. *Microbiology and Molecular Biology Reviews*.

[23] Mohamed SA, Christensen TMIE, Mikkelsen JD (2003). New polygalacturonases from *Trichoderma reesei*: characterization and their specificities to partially methylated and acetylated pectins. *Carbohydrate Research*.

[24] Cabanne C, Donèche B (2002). Purification and characterization of two isozymes of polygalacturonase from *Botrytis cinerea*. Effect of calcium ions on polygalacturonase activity. *Microbiological Research*.

[25] Pashkoulov D, Giannetti I, Benvenuto E, De Martinis D (2002). Biochemical characterization of polygalacturonases from five different isolates of *Botrytis cinerea*. *Mycological Research*.

[26] Contreras Esquivel JC, Voget CE (2004). Purification and partial characterization of an acidic polygalacturonase from *Aspergillus kawachii*. *Journal of Biotechnology*.

